# Psychiatric leadership development in postgraduate medical education and training

**DOI:** 10.1192/bjb.2021.32

**Published:** 2022-06

**Authors:** Alex Till, Radhika Sen, Helen Crimlisk

**Affiliations:** 1 Health Education England – North West, UK; 2 Royal College of Psychiatrists, UK; 3 Camden and Islington NHS Foundation Trust, UK; 4 Sheffield Health and Social Care NHS Foundation Trust, UK

**Keywords:** Leadership, management, education and training, postgraduate

## Abstract

The value of strong, compassionate medical leadership in the delivery of high-quality care to patients within mental health services is clear. Leadership development, however, is far less well explored. This article is for psychiatric trainees, trainers and mental health organisations. It provides an introduction to the importance of leadership development within postgraduate medical training, the theory that should underpin its delivery, and the opportunities for both informal and formal leadership development within psychiatric training.

Doctors are frequently the head of healthcare teams,^1^ with leadership an emergent theme within the skills, knowledge and behaviours required of our future doctors.^2^ Leadership is the most influential factor in shaping organisational culture.^3^ It must be compassionate to stimulate improvement and innovation,^4^ and is the hallmark of high-quality care.^5^

Leadership can be conceptualised as ‘a process that occurs in a group context, involving the influence of others towards the attainment of a common goal’.^6^ Yet leadership is better conceptualised as a ‘triad’, whereby leadership, management and followership are distinct, but inextricably linked skills, and hereafter are considered as one.^7,8^

Leadership has been formally recognised as a responsibility for all doctors since 2012,^9^ and is gradually being integrated into both undergraduate^10^ and postgraduate medical curricula.^11^ To help guide trainees’ development within these domains, the Faculty of Medical Leadership and Management (FMLM) have produced standards for medical professionals^12^ that clearly outline four key domains for doctors:

1. A doctor's ability to know and understand themselves and their impact on others.
2. A doctor's ability to know when to lead and follow, and how to establish and lead teams.
3. A doctor's ability to understand and positively contribute to the strategic direction and operational delivery of their organisation.
4. A doctor's ability to understand and positively contribute to the healthcare system.

Despite this initiative, the relevance and importance of leadership development is yet to be fully embraced by the medical profession. It is often still perceived as an ‘optional extra’ within medical training,^13^ and an unpopular, unrewarding and risky ‘add-on’.^14^ This lack of external validation, and outdated traditional view of medical professionalism, compromises the development of so-called ‘willing hybrids’, and the integration of leadership and managerialism as a legitimate and essential aspect of medical professionalism.^15,16^

The argument for strong medical leadership within healthcare organisations is well defined.^17,18^ Mental health services face unprecedented challenges on an almost daily basis. Doctors must be engaged in leadership^19^ and with their organisations as a whole.^20^ They must be capable of understanding and overcoming the volatile, uncertain, complex and ambiguous^21^ nature of providing not only health, but also social care, within an increasingly integrated systems-based approach.

Leadership development, therefore, is crucial, yet far less well explored. There is little robust evidence for the effectiveness of specific leadership development programmes, with a proliferation of diverse interventions throughout medical training and healthcare as a whole.^3^

It is clear, however, that the systematic integration of basic leadership development for all doctors needs to be embedded throughout postgraduate medical training, supplemented by enhanced and advanced opportunities for aspiring medical leaders, and that trainees themselves need to realise and accept leadership responsibility.

This article is for psychiatric trainees, trainers and mental health organisations. It builds on the already established position highlighting the positive impact of psychiatrists on leadership and management.^22^ It provides an introduction to the importance of leadership development within postgraduate medical training, the theory that should underpin its delivery, and the opportunities for both informal and formal leadership development available to psychiatric trainees within postgraduate medical training throughout the UK.

## Leadership development theory

Healthcare, like many other industries, often falls victim to the ‘great training robbery’.^23^ Organisations frequently support and encourage individuals to undertake externally provided leadership development programmes, and yet often fail to provide appropriate conditions on their return for them to contextualise and apply their learning within their clinical environment. Although this can be successful in developing their knowledge, skills and competencies, it effectively sets them up to fail, and leads to the continuation of entrenched and established ways of working.^24^

If exclusively developed within the ‘classroom’, development will largely remain ‘horizontal’,^25^ and leaders will fail to learn how to change and adapt to ever-changing circumstances.^23^ Alternatively, an ‘*in vivo*’ approach, with the right conditions coordinated throughout a programme, can harness ‘vertical’ leadership development through the delivery of ‘heat experiences’, ‘colliding perspectives’ and ‘elevated sense-making’.^26^

Heat experiences involve organisations taking calculated risks, and exposing leaders, who themselves must take a risk, to complex uncomfortable first-time challenges. Here, they are forced to grow, not just because they want to, but because there is a chance of failure, people are watching, and results matter. They are ‘the what’ that compels leaders to disrupt and disorient their traditional thinking styles, to discover new and better ways to make sense of the challenges they face. Caution must be excised, however, to get the ‘temperature right’, and control the heat within a safe space, with the right support, available at the right time, to help the individual stay within that learning ‘sweet spot’. Importantly, there also needs to be an appropriate level of both individual and organisational tolerance of failure.^27^

Colliding perspectives expand the number of lenses through which a leader can analyse complexity and challenge their existing mental models.^28^ Individuals are encouraged to view the world through the eyes of different stakeholders, including that of patients, carers and other members of the multidisciplinary team, and by standing in their shoes, learn to identify and manage conflicting paradigms. Ideally combined with peer coaching, partnered with different disciplines, these colliding perspectives provide ‘the who’ that is inclusive and embraces a range of diverse thought.^29^

Elevated sense-making is the final piece, ‘the how’, that leads to transformational change.^30^ Not only must structured reflection (through reflective practice groups or action learning sets) be facilitated to allow leaders to reflect on their development over time and integrate their experiences with new found perspectives, but their development should be guided by experienced coaches or mentors.

It is vital that these three conditions are provided within postgraduate medical training, whether through activities embedded informally within the work environment, or formally via a targeted approach within leadership development programmes. Although value can still be extracted from opportunities that do not provide these conditions, leadership talent will remain untapped and dormant within healthcare organisations unless these are met.^31^

## Informal leadership development

The common perception of leadership as an optional extra remains pervasive throughout the medical workforce. Combined with a ‘curriculum lag’, whereby there is a delay in implementing and adopting the latest evidence into training, the development of leadership competencies remains somewhat tokenistic.^32^ The stars are aligning, however, and the importance of leadership development in both undergraduate and postgraduate medical curricula is slowly being realised.^33^

The message is clear. Leadership development does not begin or end at any particular stage of training. Leadership is for all doctors, at every stage, and should not be postponed until doctors are formally appointed to a leadership or management position. Leadership is rather a developmental process on a lifelong continuum, with individuals nurtured to help recognise and fulfil leadership roles, especially in the earliest of stages of their careers.^34^

Everyday leadership experiences are commonplace within psychiatric clinical settings, where decision-making is complex and ethical tensions arise through divergent views, roles and responsibilities within teams.^35^ Although often undervalued, these present opportune leadership development experiences where, among a range of other activities, leadership can be developed through acute crisis situations, multidisciplinary meetings, mentoring junior colleagues, medical education, clinical governance and quality improvement projects. It is important to recognise, name and make sense of these everyday leadership experiences within training, through supervision, and allow trainees to recognise the value of ‘little l’ leadership within their teams.^36^

More formal opportunities also exist through representative roles available locally, regionally and nationally, that can all lead on to providing more enhanced leadership experiences and the three primary conditions of vertical leadership development in their own right. Likewise, voluntary and additional professional activities can provide excellent leadership experience. For example, roles available within Royal Colleges, special interest groups, trade unions, healthcare regulators, the General Medical Council, or as a governor for healthcare and affiliated organisations.

Irrespective of the particular opportunity, whether formal or informal, obtaining feedback is critical for gaining insight into the trainees’ own perception of their leadership capabilities, and the perceptions held by others of their behaviours and performance.

Integral tools within psychiatric training for facilitating such structured feedback are the Mini-Peer Assessment Tool and Direct Observation of Non-Clinical Skills (DONCS) workplace-based assessments. These are used across the General Medical Council-approved curricula for both core and specialty psychiatric training within the UK, to assess a trainees performance and allow trainees to demonstrate their leadership capabilities.^37,38^

Originally founded on The CanMEDS 2005 Physician Competency framework,^39^ DONCS are applicable to a range of diverse non-clinical skills, and structuring feedback on leadership experiences around the seven domains can at times feel convoluted and ambiguous.

Table 1 has been conceptualised from the evidence base to help suggest key competencies for psychiatric leaders within a DONCS assessment, and aims to support both trainees and trainers attaining and delivering feedback.^39–42^

**Table 1:** Your guide to a Leadership and Management DONCS

DONCS domain	DONCS descriptor
Medical expert	As a medical expert, the psychiatric leader integrates the other six intrinsic roles (as below) to negotiate complexity, uncertainty and ambiguity, while contributing to continuous improvement and maintaining the highest standards of clinical knowledge, person-centred care and professional values.
Communicator	As communicators, psychiatric leaders will develop trusted interpersonal relationships with and between individuals. They will accurately elicit, synthesise and convey relevant information, in both oral and written form, to develop a shared understanding between stakeholders of the relevant issues, problems and plans at hand.
Collaborator	As collaborators, psychiatric leaders will work effectively in partnership with patients, carers and extended multidisciplinary teams of expert professionals. This will take place in multiple locations, within and across organisational boundaries, to deliver optimal patient care and prevent, negotiate and resolve interpersonal conflict.
Manager	As managers, psychiatric leaders will participate in the improvement of healthcare delivery, manage resources appropriately, develop competence in health informatics and contribute to the effectiveness of the team, organisation and system they work within.
Health advocate	As health advocates, psychiatric leaders will promote diversity and inclusion. They will utilise their expertise and influence to identify and advance the health and well-being of individual patients, communities and populations.
Scholar	As a scholar, the psychiatric leader pursues continuous personal and professional development. They will critically evaluate information, facilitate and create a safe space for the mentoring and learning of others, and contribute to the creation, dissemination and integration of evidence-based knowledge into practice.
Professional	As a professional, the psychiatric leader engages with healthcare regulation; maintains the highest standards of personal, professional and ethical behaviour; embraces a compassionate and inclusive approach and is committed to reflective practice. They support the well-being of both themselves and the staff around them.

DONCS, Direct Observation of Non-Clinical Skills.

## Formal leadership development

There are a plethora of leadership development programmes, schemes and other opportunities available throughout the UK. This can be confusing for both trainees and trainers alike, occurring both in and out of training programmes, with a diverse range of requirements, levels of involvement and financial commitment.

Attempting to provide a brief description of these opportunities, Table 2 outlines a sample of the variety of leadership and management training that is currently available to psychiatric trainees within the UK. It is important to remember, however, that there is an almost constantly evolving stream of new opportunities depending on interests and geography, and that this table is likely to be quickly outdated.

**Table 2:** Leadership and management training availability to psychiatric trainees (correct as of 2020/21)

				Eligibility/target	Key points
Fellowships (12 months)	Outside of local education provider	National	National Medical Directors Clinical Fellow Scheme^43,^^a^	On completion of the Foundation Programme	Funded, out of programme, centrally recruited to national organisations. Apprenticeship model, developed in collaboration with UK Government and arm's length bodies, mentored by senior national leaders and undertake a range of project and policy work.
			Welsh Clinical Leadership Training Fellowship^44,^^a^	C/ST3 and above	
			Scottish Clinical Leadership Fellowship Scheme^45,^^a^	C/ST2 and above	
			Northern Ireland (NIMDTA) Achieve, Develop, Explore Programme for Trainees (ADEPT)^46,^^a^	ST4 and above	
		Local/regional	Darzi Fellowship Programme – London South Bank University^47,^^a^	C/ST3 and above	Funded, out of programme, individually recruited to local/regional organisations through NHS Jobs. Undertake London South Bank University PGCert in Leadership in Health, alongside work-based fellowship challenges.
			Future Leaders Programme – Health Education Yorkshire and Humber^48,^^a^	C/ST3 and above	Funded, out of programme, individually recruited to local/regional organisations through NHS Jobs. Undertake PGCert of their choice, alongside work-based fellowship challenges.
	Within local education provider	National	Royal College of Psychiatrists’ Leadership and Management Fellow Scheme^49^	ST4 and above	Sponsored, in programme experience, utilising special interest time, individually recruited to by local/regional organisations. Face to face (7 days, London/Liverpool), with work-based application.
			Royal College of Physicians’ (London) Chief Registrar Programme^50,^^a^	ST4 and above	Funded, in or out of programme experience, individually recruited to by local organisations. Face to face (5 days, London/Liverpool) and 40–50% protected time to practice leadership and quality improvement.
Courses and programmes		National	Royal College of Psychiatrists’ Leadership and Management for Trainees and New Consultants^51^	Available to all	Self-funded, face to face (1 day).
			NHS Leadership Academy Edward Jenner (6 weeks)^52^	Available to all	Free, online (5 h per week), with work-based application.
			NHS Leadership Academy Mary Seacole (6 months) or Rosalind Franklin (9 months)^52^	Core or higher training, respectively	Predominantly self-funded (circa £1000), sponsorship and bursaries available *ad hoc*. Online (5 h per week) and face to face (Mary Seacole 3 days/Rosalind Franklin 8 days – regional), with work-based application.
			NHS Wales 1000 Lives ‘Improving Quality Together’^53^	Available to all	Free, online (bronze) and face to face (silver, 2 days), with work-based application.
			Northern Ireland (NIMDTA) ENGAGE Clinical Leadership and Improvement Programme^54^	ST5 and above	Funded, face to face (1 day, 7 evenings).
			NHS Education for Scotland Leadership and Management Programme (LaMP)^55^	C/ST3 and above	Funded, online and face to face (2 days), with work-based application.
		Local/regional	Learning to Lead – East Midlands Leadership and management programme (3 years)^56^	On completion of the Foundation Programme	Funded, face to face (3 days), with work-based application through a multi-professional quality improvement project.
			Chief Residents’ Management and Leadership Programme – Health Education East of England^57^	ST5 and above	Funded, centrally recruited, face to face (10-day Judge Business School ‘mini-MBA’), with work-based application and supported leadership role.
Postgraduate education	Master's in Medical Leadership (MSc)		Various institutions offer ‘step-on, step-off approach’ from PGCert to PGDip to MSc (1–3 years)^58,^^59^	Available to all	Predominantly self-funded (£2500–£25 000), sponsorship and bursaries available. Part time, moderate workload, variable online versus face to face.
	Master's in Business Administration (MBA)		Various institutions, some offer healthcare specialties or NHS endorsement (2–3 years)^60,^^61^	Available to all	Predominantly self-funded (£15 000–£90 000), sponsorship and bursaries available. Part time, heavy workload, variable online versus face to face.

NIMDTA, Northern Ireland Medical & Dental Training Agency; NHS, National Health Service; PGCert, Postgraduate Certificate; MBA, Master of Business Administration; MSc, Master of Science; PGDip, Postgraduate Diploma.

a. Predominantly non-clinical (although some do allow limited ongoing clinical activity), and therefore often require an extension to training via out-of-programme experience approval.

**Fig. 1:**
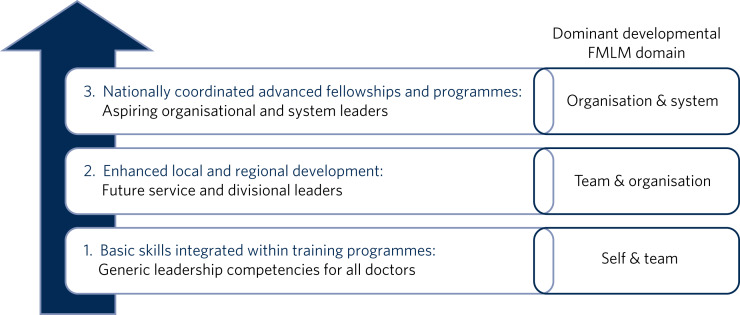
A tiered approach to leadership development. FMLM, Faculty of Medical Leadership and Management.

Such formal leadership development could be conceptualised through a tiered approach (Fig. 1). Firstly, basic generic professional capabilities are provided in leadership for all doctors via an integrated approach within local training programmes. A second tier then provides enhanced local and regional leadership development offers for future service and divisional leaders. Then finally, at the upper tier, nationally coordinated, advanced programmes and fellowships, are delivered for aspiring organisational- and system-level leaders.

## Discussion

It is important to note that leadership development does not suit a one-size-fits-all approach, and that the evidence does not suggest that any particular activity should be completed before another.^3^

Up to 90% of learning occurs informally, through often spontaneous, unstructured activities embedded within the work environment.^62^ As revealed through the developmental journeys of medical, clinical and managerial National Health Service chief executives,^63^ although formal leadership development can be transformational for some, it is insufficient in isolation.

Leadership development can often be better attributed to engagement with inspirational role models, and through the opportunistic experiences that emerging leaders seized because they could, and because they were motivated to make a difference.

For this very reason, it is vital that we overcome the shortage of role models with protected characteristics. Those in medical leadership positions must reflect the wider workforce and communities we serve.^64^ It is not permissible to allow ourselves to fall victim to a complex range of social, cultural, political, economic and historical factors, whether unconsciously or otherwise, that marginalise and disempower aspiring leaders from diverse backgrounds.

Equality and diversity should be a top priority for all individuals and organisations. We must counteract the deeply embedded prejudice and discrimination that have become endemic within modern society.^65^ No matter what the characteristic, whether it be gender, sexual orientation, race, religion or any other characteristic that differs from the majority of leaders, these individuals do not easily fit within a structure that is coded towards the ‘snowy white peaks of the NHS’,^66^ and this must be overcome.

To build this diversity into our psychiatric leadership, and that we need within our mental health services, we must embrace the ‘lived experience’ of talented leaders regardless of demographic differences, and adopt an inclusive leadership approach.^67,68^ After all, organisations with greater inclusion, rather than merely diversity, are proven to perform better, with greater improvement and innovation, higher levels of morale, and new insights that maximise the potential of employees.^68,69^

As Vernā Myers puts it ‘Diversity is being invited to the party; inclusion is being asked to dance’.^70^

Multiple strategies can be employed to improve diversity and develop an inclusive approach,^65–72^ but it is no easy task. Fundamentally, it is a cultural change. All doctors, and particularly existing leaders, must engage with these groups, create a psychologically safe space, listen to their stories, confront the hard truths laid bare by their experiences, and challenge the status quo, making diversity and inclusion a personal priority. Allies from non-disadvantaged or less discriminated against groups can confront and have a powerful impact on the behaviour of others. They must not just question what privileges they have been afforded that others might not, but question and reflect on the absence of challenges and barriers that they have not had to overcome but others might. Crucially, they must then act, working within the organisation and system to counteract and mitigate these for others.

Individuals should not feel like ‘outsiders’. We should rather recognise an individual's need to belong and proactively seek role models with greater diversity, to make the inclusion of leaders with protected characteristics explicit and visible. This allows those from marginalised groups to identify with the existing leadership, see themselves as leaders and, crucially, feel empowered to seize those opportunistic leadership experiences that are so crucial for their development. In combination, active career sponsorship will be crucial to retain and advance their leadership talent, with mentorship being a powerful mechanism for both the individual and the organisation.^68,73^

It truly is an inclusive leadership approach that is required. Demographic diversity in isolation, is insufficient. Active role-modelling and the support of key allies in existing leadership positions is essential to provide equitable access to formal and informal leadership development.

Just as we would expect within clinical practice, trainees of all backgrounds must be supported by experienced trainers who expose them to increasingly uncomfortable challenges, yet who provide them with the psychological safety net to take risks, experiment and develop ‘on the job’.

## Conclusions

Mental health services face unprecedented challenges on an almost daily basis. To survive in this world, and lead quality improvement towards more preventative, holistic and personalised care, doctors must develop a deep understanding of leadership and effectively demonstrate the core values and behaviours expected of medical professionals.

Greater attention must be paid towards medical leadership development and an inclusive approach, whereby all doctors, from every background, are supported to advance. This has never been more important. The view of leadership development being an optional extra within medicine, or a skill set to be developed later in a medical professional's career, is outdated.

No matter which one of the many diverse interventions are pursued, doctors must engage with, and be supported in, both informal and formal leadership development. This is a collective responsibility, and much more must be done to ensure equity of access to leadership development for all, from the earliest of stages in a doctor's career.

## About the authors

**Dr Alex Till** (MBChB, MRCPsych, MSc, MBA) is a Specialty Registrar in Forensic Psychiatry with Health Education England – North West, UK. **Dr Radhika Sen** (BSc, MBChB, MRCPsych) is a Consultant Psychiatrist in General Adult and Old Age Psychiatry with Camden and Islington NHS Foundation Trust, UK; and a Leadership and Management Fellow (2019/20) with the Royal College of Psychiatrists, UK. **Dr Helen Crimlisk** (FRCPsych, MSc, FAcadMEd) is Deputy Medical Director of Sheffield Health and Social Care NHS Foundation Trust, UK; an Associate Registrar for Leadership and Management with the Royal College of Psychiatrists, UK; and a Generation Q Fellow with The Health Foundation, UK.
